# General practice responses to opioid prescribing feedback: a qualitative process evaluation

**DOI:** 10.3399/BJGP.2020.1117

**Published:** 2021-08-24

**Authors:** Su Wood, Robbie Foy, Thomas A Willis, Paul Carder, Stella Johnson, Sarah Alderson

**Affiliations:** Leeds Institute of Health Sciences, University of Leeds, Leeds.; Leeds Institute of Health Sciences, University of Leeds, Leeds.; Leeds Institute of Health Sciences, University of Leeds, Leeds.; West Yorkshire Research & Development, NHS Bradford District and Craven CCG, Bradford.; West Yorkshire Research & Development, NHS Bradford District and Craven CCG, Bradford.; Leeds Institute of Health Sciences, University of Leeds, Leeds.

**Keywords:** analgesics, opioid, feedback, general practice, Normalisation Process Theory, prescribing, process evaluation, qualitative research, quality and safety

## Abstract

**Background:**

The rise in opioid prescribing in primary care represents a significant public health challenge, associated with increased psychosocial problems, hospitalisations, and mortality. An evidence-based bimonthly feedback intervention to reduce opioid prescribing was developed and implemented, targeting 316 general practices in West Yorkshire over 1 year.

**Aim:**

To understand how general practice staff received and responded to the feedback intervention.

**Design and setting:**

Qualitative process evaluation involving semi-structured interviews, guided by Normalisation Process Theory (NPT), of primary care healthcare professionals targeted by feedback.

**Method:**

Participants were purposively recruited according to baseline opioid prescribing levels and degree of change following feedback. Interview data were coded to NPT constructs, and thematically analysed.

**Results:**

Interviews were conducted with 21 staff from 20 practices. Reducing opioid prescribing was recognised as a priority. While high achievers had clear structures for quality improvement, feedback encouraged some less structured practices to embed changes. The non-prescriptive nature of the feedback reports allowed practices to develop strategies consistent with their own ways of working and existing resources. Practice concerns were allayed by the credibility of the reports and positive experiences of reducing opioid prescribing. The scale, frequency, and duration of feedback may have ensured a good overall level of practice population reach.

**Conclusion:**

The intervention engaged general practice staff in change by targeting an issue of emerging concern, and allowing adaption to different ways of working. Practice efforts to reduce opioid prescribing were reinforced by regular feedback, credible comparative data showing progress, and shared experiences of patient benefit.

## INTRODUCTION

Healthcare systems internationally are attempting to counter rises in opioid prescribing.^1^^–^^3^ Such rises are largely attributed to prescribing for chronic non-cancer pain,^4^ where opioids are of limited effectiveness and can cause dependence.^5^ Prescribed opioids — even weaker opioids such as codeine — are associated with increased psychosocial problems, hospitalisation, and mortality.^6^^–^^7^

The rise in opioid prescribing for chronic pain in UK primary care is well documented.^8^^–^^9^ Although this rise may have peaked,^10^ one study has shown that almost one in 20 adults are prescribed long-term opioids.^8^ Prescribing rates are higher in areas of greater social deprivation.^11^ However there is substantial, poorly explained variation among practices, suggesting that much opioid prescribing is driven by GP habits and norms rather than patient need. Patients and GPs are unsatisfied with opioid prescribing in chronic non-cancer pain.^12^ Patients experience repeated consultations that fail to meet their needs for pain relief, adequately explain their symptoms, and improve their quality of life. GPs recognise the shortcomings of prescribing and negotiating alternative approaches to treatment, but have limited access to alternative sources of support.^12^ Given accumulating evidence of harm, reducing opioid prescribing for chronic pain would deliver substantial population benefit.

The authors responded to a request for help by clinical commissioning groups (CCGs) in West Yorkshire by designing and delivering a year-long audit and feedback intervention, the Campaign to Reduce Opioid Prescribing (CROP) (TIDieR Statement available^13^). Audit and feedback is a widely used approach that aims to improve patient care by reviewing healthcare performance against explicit standards and encouraging action to address discrepancies between desired and actual practice. Although effect sizes are generally modest,^14^ it offers an ‘upstream’ population approach compared with interventions targeting individual patients; for example, holistic pain management.^15^ Feedback may prompt GPs to think more cautiously when considering opioid prescribing (especially for patient groups at higher risk of transition to long-term use, or for stronger opioid prescribing)^8^ and encourage medication reviews for patients who may not benefit. In a controlled interrupted time series analysis, it was found that repeated feedback over 1 year significantly reduced opioid prescribing — particularly in high-risk patient groups — without increasing prescriptions for other analgesics, saving around £700 000 in predicted opioid prescribing costs over the intervention year.^13^

**Table:** How this fits in

There is international concern about rising opioid prescribing, mostly for chronic non-cancer pain where population harms are likely to outweigh benefits. An audit and feedback intervention reduced opioid prescribing in UK primary care; however, it is not known how general practices receive and respond to feedback. This study found that practices generally welcomed non-judgemental comparative feedback addressing an issue of emerging concern, which allowed them to respond flexibly in mounting a range of organisational and clinical actions. Despite growing moves towards use of electronic clinical dashboards for accessible and continually updated data, general practices still value simpler formats of feedback to enable sharing within teams.

How practices received and responded to CROP within the routines, systems, and constraints of primary care was evaluated, as was the degree to which changes became embedded in practice.

## METHOD

### Design and setting

West Yorkshire in England has a socioeconomically and ethnically diverse population of approximately 2.2 million residents.^16^ There were 317 general practices organised within 10 CCGs in 2016. Qualitative, semi-structured interviews were conducted with primary care professionals responsible for prescribing and quality improvement. The process and experience of receiving feedback on opioid prescribing was explored. Interviews took place 14–19 months after the final feedback, allowing exploration of both perceived initial and sustained impacts. COREQ informed the methodology and reporting.^17^

### Intervention

Between April 2016 and March 2017, a total of six bimonthly feedback reports were delivered (via emailed PDF and post to the practice manager) to 316 of 317 general practices (one practice abstained from data sharing). Practice-specific, comparative feedback reports presented overall opioid prescribing as well as that for specific groups at risk of long-term or strong opioid prescribing (see Supplementary Figure S1 for the example report). Patients with coded cancer, palliative care, or drug dependence were excluded.^8^ Evidence-informed behaviour change techniques were embedded to enhance effectiveness.^18^ These included use of peer comparison, feedback on behaviour, emphasising the credible source of feedback, goal setting, and problem solving. The feedback advocated reducing opioid prescribing and suggested options for action, but was ‘non-prescriptive’, as responses were at practices’ and prescribers’ discretion. Practices were also provided with access to electronic health record searches, allowing them to identify and review individual patients.

### Theoretical framework

Data collection and analysis was informed by Normalisation Process Theory (NPT), a theory of implementation designed to assist interpretation of how people in healthcare teams individually or collectively embed (or not) new work practices.^19^^–^^20^ It includes four constructs:

1. Coherence;
2. Cognitive participation;
3. Collective action; and
4. Reflexive monitoring.

NPT constructs were used as sensitising devices to enquire about how general practices responded to and acted on the feedback (Box 1).^20^^–^^21^

**Box 1.** Normalisation Process Theory constructs and components^20^ interpreted for the data collection and evaluation of CROP

**NPT constructs**	**NPT components and interpretation for CROP**			
**Coherence**	**Differentiation**	**Communal specification**	**Individual specification**	**Internalisation**
How do participants understand and attribute value to CROP?	How does CROP differ with what is currently done in practice?	Does the practice team have a shared understanding of CROP?	What work has been done individually to understand CROP?	Has the practice attributed worth to the CROP project?

**Cognitive participation**	**Initiation**	**Enrolment**	**Legitimation**	**Activation**
Enrolment and engagement of individuals and groups	What, if any, were the key drivers for engagement with CROP?	How were others persuaded to take part? Was there buy-in?	How have they ensured CROP fits with values and ways of working?	What has been done to sustain change in practice?

**Collective action**	**Interactional workability**	**Relational integration**	**Skill set workability**	**Contextual integration**
Organising and doing the work	How was CROP operationalised?	How was confidence and accountability built and maintained?	Who did the work, and how was it allocated?	What resource work was done to enact CROP?

**Reflexive monitoring**	**Systematisation**	**Communal appraisal**	**Individual appraisal**	**Reconfiguration**
Reflecting on progress and making necessary adjustments	How has it been determined how effective CROP is in practice?	What group evaluation has there been of the worth of CROP?	How has CROP affected them as an individual, and impact on other work?	Has the team redefined or modified practices after appraisal?

*CROP = Campaign to Reduce Opioid Prescribing. NPT = Normalisation Process Theory.*

### Sampling and recruitment

Intervention practices were purposively sampled using a 2 × 2 sampling frame of baseline opioid prescribing and change in prescribing during the intervention (Table 1). Invitations were emailed to practices, with the aim of recruiting around five from each group to achieve a total of approximately 20 practices. Recruitment was monitored against data saturation, and the stopping criterion was set as when three consecutive interviews elicited no new themes or insights.^22^ Practices were asked to nominate interviewees. Each participating practice was offered £100 in recognition of opportunity costs.

**Table 1.** Number of practices recruited from the 316 intervention practices using the 2 × 2 sampling frame based on initial opioid prescribing, and change in prescribing over the intervention year

		**Change in prescribing**	

		**Above average reduction**	**Below average reduction or increase**
Initial prescribing	Above average	4	4
	Below average	6	6

### Qualitative data collection

All interviews were conducted by a post-doctorate pharmacist researcher with primary care and qualitative research experience, using an NPT-informed topic guide (Supplementary Figure S2). Practice feedback reports were reviewed with participants at interview to help recall and reflection. Interviews were audio-recorded and transcribed verbatim before analysis. Field notes were made immediately after each interview.

### Data analysis

Data were collected and analysed inductively in the light of NPT,^20^ and thematic analysis was used to identify recurring themes across the data and examine relationships between themes.^23^ While NPT guided the analysis, it remained open to other notable findings that did not fit within this framework. Two authors independently coded four transcripts for emerging themes, one from each sample group, and then compared coding and resolved discrepancies. The remaining transcripts were coded independently, and overarching themes generated by combining and comparing codes.

How codes related to each other was mapped, and negative cases were looked for to examine similarities and differences within and between how practices received and acted on feedback reports. Further analysis was conducted by charting themes in Excel to enable review of practice characteristics, opioid prescribing levels, and their contribution to themes, and identification of when the stopping criterion had been met for data saturation. The data analysis was completed before the results of the interrupted time series analysis were known.

### Patient and public involvement

The project was developed in consultation with the existing primary care research patient and public involvement panel.^24^ Two panel members helped to refine the research question and protocol, including by adding a topic guide item on involvement of patient participation groups in practice quality improvement.

## RESULTS

From 148 invitations, 20 practices responded and were interviewed, representing all sample frame groups (Table 1).

Data saturation was reached after 17 interviews, as no new themes were elicited from three subsequent interviews (Supplementary Figure S3). A second person joined one interview, making 21 interviewees: 12 GPs, four practice managers, three practice pharmacists, one operations manager, and one IT manager, from nine out of 10 CCGs. Interviews lasted 30–40 minutes and all were conducted in general practices. Supplementary Table S1 summarises practice characteristics and reported actions on opioid prescribing.

Most practices reported actions to reduce opioid prescribing, including those that did not reduce opioid prescribing during the intervention. This analysis suggested no indication of a relationship between NPT components and practice characteristics (that is, list size, role, initial opioid prescribing level, and changes in prescribing).

Supplementary Table S2 summarises the coding under NPT components and illustrates the wide range of practice responses to feedback. The findings are then presented under the themes in the text. NPT highlighted five themes: deciding to act, engaging the team, flexibility in responding, overcoming challenges, and realising benefits.

Two further themes were identified: getting the intervention into general practices and feedback report format.

### Deciding to act

The feedback intervention was reported as generally well received, with recognition that reducing opioid prescribing was a clinical priority. Most participants said they had not considered acting on opioid prescribing previously, while those who had suggested that the feedback helped motivate and focus effort:

*‘Although we were relatively low in the figures, it felt like we weren’t doing any rational prescribing and it’s important.’*

(Participant [P]1 [GP, lower prescribing practice, reduced prescribing])

Participants reported that reviewing feedback and finding high doses of prescribed strong opioids, against a background of ongoing media coverage of opioid-related harm, highlighted the need for action. Graphical practice comparisons and observing visible rates of change were perceived to reinforce action. They were able to report examples of patient benefit from reducing and stopping opioids.

Some expressed initial concerns about the process, particularly about conflict between drivers of clinical excellence and patient satisfaction. One participant felt that GP overload with competing priorities was a factor in their practice not engaging with feedback:

*‘There are so many different pushes on so many different areas. It really has got I believe completely silly! I think you can change behaviour, but you can’t change behaviour when you’re changing 20, 30, 40 things at the same time.’*

(P16 [GP, lower prescribing practice, no change in prescribing])

### Engaging the team

Interviewees described the feedback intervention as not only being readily assimilated in practices with a clear structure for quality improvement, but also as encouraging less structured practices to make changes. They felt the need for enthusiastic leadership, citing different team members as taking the lead:

*‘It’s finding the enthusiast, yes, it needs somebody that can push it and repeat it and have ownership of it.Who wants to be opioid champ? But it needs somebody to encourage, to bully, to run the audit. To educate the team to continue the downward pressure on reducing it and not initiating it and looking at alternatives and making everybody opioid aware.’*

(P1 [GP, lower prescribing practice, reduced prescribing])

Responses suggested that a single motivated staff member in a small practice led effective implementation, while in larger practices a shared vision and joint working were important. Also, the content of feedback reports helped engage staff when discussed at team meetings. Examples of team engagement reported included identifying ‘quick wins’ to encourage action, selected GPs trialling approaches with patients initially and reporting back, and instigating training.

The feedback reports were perceived to provide a clear presentation of evidence, with the bimonthly delivery, and evidence of change in prescribing reinforcing confidence. Practices reported committing to prescribing policy changes, despite barriers such as patient resistance or resource constraints.

Participants shared examples of processes to sustain changes, including agreement of new practice policies, cessation of repeat prescribing of opioids for non-cancer pain, incorporating the feedback into educational sessions, and use of long-term locums who could attend clinical meetings:

*‘I think everyone’s been quite receptive to be honest. Because we knew that things were going to have to change. Now we’ve got long-term locums so we don’t use as many* ad hoc *if we can help, which helps because they come to the clinical meetings.’*

(P5 [Practice manager, higher prescribing practice, reduced prescribing])

### Flexibility in responding

Interviewees reported developing strategies that fitted with existing resources and ways of working, in response to the non-prescriptive reports. These included utilising practice pharmacists, conducting their own searches, or producing patient leaflets (see Supplementary Table S2 for details):

*‘We’ve had our own leaflets made up. Really educating patients. We’ve had them done in colour so it actually, patients know that it’s not just a little printed sheet and we had it done in five languages.’*

(P5 [Practice manager, higher prescribing practice, reduced prescribing])

Some practices reported identifying further resources to fit their needs, for example, published guidelines for opioid reduction,^25^ or online patient self-help tools.^26^

While GPs were considered responsible for taking most action, proactively or opportunistically, other staff in the team such as pharmacists, administrators, data managers, physiotherapists, drug counsellors, and health trainers were involved.

Some participants reported that active work to reduce opioid prescribing commenced later in, or after, the intervention period. Three participants ran the patient searches at the time of interview and found that their practice had reduced opioid prescribing after the intervention year:

*‘So we are at 41 now, from 120! We were 120* [patients prescribed an opioid] *at the end of that* [intervention] *year.’*

(P17 [GP, higher prescribing practice, no change in prescribing])

### Overcoming challenges

Participants identified risks that they mitigated by planning. Reported barriers to change included professional differences of opinion, competing pressures, large patient numbers, and the absence of any financial incentives or penalties:

*‘We actually had a couple of clinical meetings where it got quite heated unexpectedly! Around strong opiates. A couple of prescribers thought even the bottom quartile was far too high! So we took all views. We developed a policy and procedure around strong opiate prescribing for the practice.‘*

(P2 [Practice manager, lower prescribing practice, no change in prescribing])

Implementation was acknowledged as taking time and effort. However, participants reported they made change more manageable by, for example, staggering reviews to manage workload or focusing on single high-risk patient groups.

Patient expectations and resistance to change, or risking harming the patient relationship, were not considered reasons to abandon new policies. In fact, interviewees suggested that patients were often more accepting of the changes than anticipated:

*‘I think there were ones* [patients] *that just blank refused, and we had difficult conversations with — they were definitely the minority. But the majority were pretty open to it!’*

(P15 [GP, higher prescribing practice, no change in prescribing])

The feedback was considered helpful in justifying the resources required for extra appointments, clinical meetings, and the time to complete audits.

Reports were used as consultation aids. One practice reported working with a drug counsellor to help patients reduce opioid use; another referred patients to a local meditation group.

Some practices expressed a wish for further support, including a CCG-funded pharmacist, addiction management, and useful phrases to use in consultations.

### Realising benefits

Bimonthly reports were perceived as allowing practices to regularly evaluate progress:

*‘Looking at reports and then we actually implemented our action plans and we got a minus 14%!’*

(P11 [IT manager, higher prescribing practice, reduced prescribing])

*‘Good reduction. Wow. Nice … And I know we’ve put a lot of work into it. And across the three practices, I did a lot. But I know that here we really hammered it.’*

(P8 [Practice manager, higher prescribing practice, reduced prescribing])

Practices reported continuing to review opioid prescribing beyond the intervention period, spurred on by recognised improvements in high-risk groups (reducing prescribing of strong opioids and prescribed doses of all opioids). Their teams valued tangible evidence of their efforts. Some reported using responses to feedback as examples of quality improvement for Care Quality Commission (CQC) inspections:

*‘This has obviously helped us to project it to other staff members that, look this is the difference. And we need to keep doing it. That’s one of the things which we produced to CQC as well.’*

(P10 [GP, lower prescribing practice, reduced prescribing])

One participant described the feedback as initiating behaviour and culture change. Sharing cases where reducing opioids had improved patients’ lives made their team ‘passionate’ about the work (Box 2). Some participants considered that the feedback had permanently changed their prescribing behaviour.

**Box 2.** Case example given by P20 (GP)

*‘So I stillremember one lady I was seeing on a weekly basis actually, she was on MST [morphine sulphate tablets slow release], tramadol, modified release as well as tramadol acute, pregabalin, and one more drug. She had MS [multiple sclerosis] and she had really bad phases when she got on these drugs and never, somebody never took her off! And she just got used to them.* *I said: “You’re very stable now, I can’t say when you’ll relapse again, so if you relapse we don’t have any more to give you. You’re just looking at a downward spiral here. So if we can, while you’re feeling better, if you can gradually take a few things off or reduce them, we’ve got option to actually use them in the future for, for a relapse, or something.“* *And she thought on it and we started off with just taking off MST, gradually so, very gradual reduction. So I was seeing her regularly, if she relapses or she’s very anxious about it. But then as she built up that rapport with me would mean so em … she stopped MST. Stopped! I said, ‘OK fine let’s give it a break for a couple of months and then restart.’ It was coming to winter pressures time so I felt like let’s focus on core business, but we will continue this.* *She came back in spring to me and she said, “I’ve come off tramadol as well!” She had learned the principles and she did it! So I was really impressed.* *So now a couple of times when she’s come to see me, she’s said, “Look I’m a changed person! I can focus on things! I can … I want to do stuff. I’ve got that enthusiasm. Otherwise I was just a blank person!”* *So that was quite interesting. Similarly there are quite a few cases where a few of us can quote patients where it’s made a big difference in their lives so yes, it makes a big difference. So we’re quite passionate about it.*

While some practitioners feared being judged, and patients returning in pain, they expressed satisfaction in helping patients reduce their opioid use.

Participants expressed disappointment whenever opioid prescribing increased despite their efforts, but accepted that reductions in prescribing would take time to show impacts.

### Practice gatekeepers

Reports did not always reach targeted staff: some participants mentioned missing or unseen reports, as consequences of filtered practice communications or the sheer quantity of communications received.

One participant described an ‘accidental’ find on a coffee table, while others did not notice the reports until the third or fourth communication:

*‘So it was probably opened by one of the receptionists or our admin clerks. And they thought bin or common room? Bin or common room? So initially it didn’t, but when a second or maybe a third came, I probably saw three or four of them and I began to pick them up.’*

(P1 [GP, lower prescribing practice, reduced prescribing])

### Preferences for report formats

Some participants preferred paper copies of reports as these allowed sharing and discussion at practice meetings. It was easier to flick through paper reports, and large headings eased identification of different sections. Other participants preferred emailed PDF copies or to have both options, particularly for facilitating sharing in larger teams:

‘[GPs are] *so busy with everything I think when you’ve got something so clear, it’s like look! We’re there! Therefore we need to do something, I think that makes it easy.’*

(P5 [Practice manager, higher prescribing practice, reduced prescribing])

Some participants described accessing the searches to help identify the patients behind the figures. Repeated feedback was described as reinforcing impact, with many requesting ongoing updates after the end of the project. The perceived authoritative content of reports lent credibility and supported discussions on reduction with patients.

Participants appreciated the tone of the reports, highlighting the positive messages and the ‘well done’ when they had improved:

*‘I don’t think there’s anything in the way it was written or presented that made me think that I don’t want to do this.’*

(P6 [GP, lower prescribing practice, reduced prescribing])

Similarly, when prescribing had not fallen, participants generally described being encouraged by the messages that change might take time rather than being criticised. Nevertheless, one interviewee felt that more ‘sticks’ should have been used than ‘carrots’.

## DISCUSSION

### Summary

This study describes how a feedback intervention targeting opioid prescribing triggered a range of responses by general practices, and largely achieved its goals. NPT was found to be useful in understanding these responses. Practices understood that the feedback focused on patient safety rather than cost, and generally found it credible (coherence). Feedback appeared to be well received and motivating, enabling practices to respond in ways that fitted within existing resources and quality improvement processes (cognitive participation).

While all practices received feedback, their responses and subsequent actions appeared to vary considerably, with no identical responses. Practice teams that met regularly and had structured quality improvement processes and a committed lead appeared to respond best. However, certain aspects of feedback, such as suggested action plans, helped practices with weaker arrangements (collective action). Some practices only started to make changes following repeated feedback. Practice efforts to reduce opioid prescribing were reinforced by regular comparative and supportive feedback showing progress and shared experiences of patient benefit (reflexive monitoring).

The feedback reports did not consistently pass practice ‘gatekeeping’ processes and were sometimes lost among many other communications competing for attention. However, the scale, frequency, and duration of the feedback intervention probably ensured sufficient practice population reach.

### Strengths and limitations

There is now an abundant literature describing problematic opioid prescribing in general practices.^1^^–^^4^^,^^6^^,^^8^^–^^9^ To the authors’ knowledge, this is the first qualitative study of a successful approach supporting practices to reduce opioid prescribing. Perspectives were gathered from initial higher and lower prescribing practices, as well as from those that did or did not achieve reductions. The study’s use of theory illuminated how feedback was assimilated within practices. The analysis also demonstrated thematic saturation.

Study limitations are acknowledged. First, interviews took place at least a year after the intervention ended, and therefore were prone to recall bias. However, this allowed exploration of the degree to which any impacts on practice processes had become normalised, as well as the identification of later responses. Second, study participants were self-selecting, thereby probably underrepresenting those more resistant to change. The purposive sampling included practices that did not change prescribing; one participant did not recall receiving any feedback reports.

Third, the evaluation relied on reported rather than observed changes, and could not assess the numbers of practices that took deliberate action following feedback. However, participants provided evidence of action, such as examples of new protocols, action plans, or patient information. Fourth, the relative impacts of the different feedback components were not explored. However, interviewees did refer to several behaviour change techniques without prompting, such as peer comparisons, social influence, and action planning.

Finally, the feedback campaign sought a general reduction in opioid prescribing based on mounting epidemiological evidence of harm. Some reductions in prescribing may have caused distress to individual patients. However, no interviewees suggested that the feedback goals were inappropriate. Prescribing decisions were ultimately left to clinicians to negotiate with individual patients.

Participants reported surprise at the acceptance of change by many patients, and patients reporting benefits from reducing opioids were a motivating factor.

### Comparison with existing literature

Patients and GPs struggle with opioid prescribing,^12^ suggesting involvement of complex behaviours, including patient engagement.^27^ This feedback intervention engaged practices in undertaking complex actions such as gradual dose reduction for high opioid users, entailing multiple appointments and patient engagement.^28^

Audit and feedback is modestly effective in changing a wider range of clinical behaviours,^29^^–^^31^ with a median 4.3% absolute improvement in processes of care (for example, prescribing and test ordering). However, there is wide variation in effectiveness, with one-quarter of studies having no or even negative effects. This intervention drew on evidence-based suggestions for optimising feedback effectiveness,^32^^–^^33^ and a previous implementation package that reduced risky prescribing but did not improve type 2 diabetes control, blood pressure control, or anticoagulation for atrial fibrillation.^34^^–^^35^

For some targeted behaviours, practices may feel ‘overwhelmed’ by the scale of work needed and demotivated by the apparent lack of improvements in response to feedback.^35^ It was found that practice teams perceived and exerted control over goal setting and action planning in reducing opioid prescribing. The Effective Feedback to Improve Primary Care Prescribing Safety (EFIPPS) study also showed a clinically important reduction in targeted prescribing by using evidence-based behavioural change techniques to enhance responses to feedback.^36^

Most actions reported in practices that successfully reduced prescribing were at organisational level. Changes in organisational behaviours may augment improvement through a wider, more systematic reach than changes in individual clinician behaviour.^37^ Interventions are also more likely to succeed if recipients feel like they ‘own’ the feedback, rather than it being imposed.^37^ The present intervention may have enhanced ownership by allowing practices to respond in ways that fitted within their resources and ways of working.

Feedback is often used in combination with other interventions. For example, the Data-driven Quality Improvement in Primary Care (DQIP) study added educational visits and financial incentives to prescribing feedback.^38^ There were indications that the education visit was useful, but not essential, although it is difficult to disentangle the effects of individual components.^39^ The present study found that feedback alone engaged practices in action in the study’s specific context.

Individual general practices vary substantially in their ability to respond to changes in recommended prescribing.^40^ This study found that some practices acted quickly to implement changes, while others reported only noticing and acting on feedback reports later or after the intervention period. It is therefore important to consider possible lag effects of interventions when assessing their full impact.

### Implications for research and practice

Given the widespread rise in opioid prescribing, difficulties in predicting which patients will respond to opioids, and increasing evidence of harms,^41^ this study suggests feedback interventions can encourage judicious prescribing and monitoring. The intervention was well received and allowed a range of legitimate responses according to practice contexts and resources. When delivering feedback, careful attention to engagement of practice gatekeepers may increase subsequent engagement and fidelity. External coverage of trends and harms were cited as either encouraging practices to begin responding to feedback, or motivated continuation. This study suggests the wider acceptability and perceived value of feedback, targeting areas of emerging or established concern beyond opioid prescribing.

Practices generally welcomed the paper and emailed report formats that enabled subsequent sharing. There are growing moves towards use of electronic clinical dashboards for feedback that allow accessible and continually updated data. Dashboards may improve adherence to recommendations and improve patient outcomes but may also cause user fatigue, inadvertently increase barriers to data access (for example, if password protected), and may fall out of use without regular prompts.^42^

This evaluation took place under ‘real world’ conditions, increasing confidence in generalisability to routine general practice settings. There is scope for enhancing feedback effectiveness by optimising the content, format, and delivery of feedback, although there is continuing uncertainty on how best to optimise feedback in routine service development.^43^ Further effectiveness studies with parallel process evaluations are needed, to optimise feedback components and to clarify the contexts in which feedback is most likely to work.
